# Hypertrophic Cardiomyopathy: An Overview of Genetics and Management

**DOI:** 10.3390/biom9120878

**Published:** 2019-12-16

**Authors:** Polakit Teekakirikul, Wenjuan Zhu, Helen C. Huang, Erik Fung

**Affiliations:** 1 Division of Cardiology, Department of Medicine and Therapeutics, Faculty of Medicine, The Chinese University of Hong Kong, Hong Kong, China; 2 Lui Che Woo Institute of Innovative Medicine, The Chinese University of Hong Kong, Hong Kong, China; 3 Centre for Cardiovascular Genomics and Medicine, The Chinese University of Hong Kong, Hong Kong, China; 4 Division of Medical Sciences, Department of Medicine and Therapeutics, Faculty of Medicine, The Chinese University of Hong Kong, Hong Kong, China; 5 Department of Medicine (Cardiology), University of California, Los Angeles, CA 90095, USA; 6 Laboratory for Heart Failure + Circulation Research, Li Ka Shing Institute of Health Sciences, Prince of Wales Hospital and Gerald Choa Cardiac Research Centre, The Chinese University of Hong Kong, Hong Kong, China

**Keywords:** hypertrophic cardiomyopathy, inherited cardiomyopathy, genetics, gene mutation, sarcomere

## Abstract

Hypertrophic cardiomyopathy (HCM) is a genetically heterogeneous cardiac muscle disorder with a diverse natural history, characterized by unexplained left ventricular hypertrophy (LVH), with histopathological hallmarks including myocyte enlargement, myocyte disarray and myocardial fibrosis. Although these features can cause significant cardiac symptoms, many young individuals with HCM are asymptomatic or mildly symptomatic. Sudden cardiac death (SCD) may occur as the initial clinical manifestation. Over the past few decades, HCM has been considered a disease of sarcomere, and typically as an autosomal dominant disease with variable expressivity and incomplete penetrance. Important insights into the genetic landscape of HCM have enhanced our understanding of the molecular pathogenesis, empowered gene-based diagnostic testing to identify at-risk individuals, and offered potential targets for the development of therapeutic agents. This article reviews the current knowledge on the clinical genetics and management of HCM.

## 1. Introduction

Hypertrophic cardiomyopathy (HCM) is the most common heritable cardiovascular disorder characterized by left ventricular hypertrophy (LVH) that is unexplained by abnormal loading conditions, with myocyte hypertrophy and disarray, and increased myocardial fibrosis as key histopathological hallmarks [1]. These features can lead to impaired diastolic function, left ventricular outflow track obstruction (LVOTO), and cardiac arrhythmias, resulting in significant cardiac complications. The prevalence of HCM is approximately 1:500 in the general adult population [2]. HCM is mainly inherited as an autosomal dominant trait, caused by variants in sarcomere protein genes. However, its clinical heterogeneity and diverse phenotypic expression raise the possibility of nongenetic or environmental factors, that may modify the phenotype of HCM but are not yet fully characterized.

Genetic discoveries have improved our understanding of the molecular basis of HCM and ushered in the era of clinical genetic testing. Advances in next-generation sequencing (NGS) technologies allow faster and increasingly affordable gene-based diagnostics, that may truncate the diagnostic odyssey and differentiate HCM from phenocopies. The ongoing challenge includes the incomplete understanding of genetic variants in HCM genes, hampering the annotation and interpretation of genetic findings. Despite the challenges, the critical benefit provided by genetic testing has great implications for the early identification of at-risk individuals prior to the onset of clinical disease. This capability allows a unique opportunity to develop groundbreaking therapeutic agents, that can prevent or modify disease pathology by leveraging insights into myocyte biology and pathogenetic mechanisms of HCM. This review provides an overview of HCM, focusing on current knowledge about clinical genetics and management.

## 2. HCM Phenotypes

### 2.1. Clinical Phenotypes 

The clinical manifestations of HCM are highly variable. Individuals with HCM can present with a constellation of symptoms, including exertional dyspnea, fatigue, palpitations, lightheadedness, syncope, atypical chest pain, and sudden cardiac death (SCD), resulting from ventricular diastolic dysfunction, cardiac arrhythmias and LVOTO as main pathophysiologic conditions [3,4]. Signs and symptoms of HCM do not necessarily correlate with the extent or severity of LVOTO, or the degree of LVH, and a significant proportion of young patients with HCM remain asymptomatic or minimally symptomatic throughout life [5]. HCM is the most common cause of SCD in young adults and often athletic individuals, which is the most devastating complication that may occur as the first disease presentation [6,7]. The triggers that precipitate acute life-threatening arrhythmia or SCD remain poorly understood, but anatomic obstruction and electrophysiologic disturbance are important considerations. Hence, risk stratification for SCD relies on a prior history of cardiac arrest or sustained ventricular tachycardia (VT), unexplained syncope, family history of sudden death presumably caused by HCM in one or more first-degree relatives, and documentation of a maximum LV wall thickness ≥30 mm, non-sustained VT and abnormal blood pressure response during exercise [5]. 

The development of heart failure (HF)—a clinical syndrome of exaggerated exertional dyspnea in HCM patients—is disproportionate to, or occurs in, the absence of volume overload and pulmonary congestion as seen in typical HF patients. It may occur with intravascular volume depletion, impaired filling (e.g., diastolic dysfunction), arrhythmia (e.g., tachyarrhythmia), or impaired myocardial function secondary to ischemia (from oxygen supply–demand mismatch) and small-vessel coronary artery disease [4,8]. LVOTO is a fundamental feature resulting from a dynamic increase in mechanical impedance, exacerbated by the systolic anterior motion of the mitral valve, that may engender acute or intermittent symptoms of HF [4].

Arrhythmias including atrial fibrillation (AF) and ventricular tachycardia can precede the development of heart failure (HF), or complicate HF in HCM. An extensive literature review estimated that 1 in 3 to 1 in 5 patients with HCM had AF—a major risk factor for thromboembolic stroke [3,9,10]. The prevalence of AF in HF was stratified by the New York Heart Association (NYHA). The functional class increases from <10% in patients with NYHA I to approximately 50% in those with NYHA IV [11]. The current understanding is that left atrial dilatation, remodeling, and the expression of mutated proteins are potential mechanisms that sustain AF in HCM [3].

### 2.2. Pathological Phenotypes

HCM is characterized by several patterns of LVH which may be asymmetric or symmetric. HCM may variably affect any location of the LV, but classically involves the thickening of the basal anterior septal wall, obliterating the LVOT. Correlations between genetic etiology and hypertrophic morphologies in HCM remain incompletely understood [12]. The main histopathological hallmarks of HCM are myocyte hypertrophy and disarray, and myocardial fibrosis [1,3]. However, myocyte disarray has also been identified in other forms of cardiac disease. Small intramural coronary arteries with arterial dysplasia is observed in individuals with HCM, leading to clinical ischemia and replacement fibrosis [13].

## 3. Genetics of HCM

HCM is a genetically heterogenous disease with variable expressivity and incomplete penetrance [1,3,14,15]. The phenotypic variability is due, in part, to the causal variant exerting effects in concert with several other genetic and nongenetic factors. An autosomal dominant trait of HCM has long been appreciated [16]. Linkage analysis of large HCM families led to the identification of disease-causing genes in different contractile apparatus components [17]. Subsequent genetic studies revealed multiple separate pathogenic variants in sarcomere protein genes in approximately 40%–60% of patients with HCM, collectively establishing HCM as a disease of sarcomere proteins [14,15]. Of those patients with positive genetic testing, most disease-causing variants occur in myosin heavy chain (*MYH7*) and myosin binding protein C (*MYBPC3*). In 5%–10% of cases, HCM is caused by mutations in non-sarcomere genes that are associated with neuromuscular disease (e.g., Friedreich ataxia), metabolic disorders (e.g., Barth syndrome) or genetic syndromes (e.g., Noonan syndrome) [18].

### 3.1. Cardiac Sarcomere

The cardiomyocyte contains bundles of myofibrils that represent the fundamental contractile units and are composed of thick myosin and thin actin filaments, closely associated with the regulatory troponin complex, α-tropomyosin, and cardiac myosin binding protein C [19]. The depolarization of myocytes activates the L-type voltage-gated calcium (Ca^2+^) channels on the membrane, causing a Ca^2+^ influx into the cell, triggering the release of stored Ca^2+^ from the sarcoplasmic reticulum via the cardiac ryanodine receptor channels. Increased intracellular Ca^2+^ binds to troponin C, inducing a conformational change in the troponin complex (troponin I and troponin T) and displacing tropomyosin from the myosin-binding sites of actin. As a result, myosin binds actin, allowing the formation of cross bridges, and ATP hydrolysis occurs to release energy to propel myosin sliding along the actin filament. The binding of new ATP results in myosin being released from actin, and Ca^2+^ being pumped back into the sarcoplasmic reticulum through an ATPase-dependent pump or extruded via the sodium–calcium (Na^+^/Ca^2+^) exchanger. Troponins I and T and tropomyosin rebind actin, shielding the myosin-binding sites and resulting in muscle relaxation [1].

### 3.2. Sarcomere Gene Mutations in HCM

Pioneering studies by Drs. Christine and Jonathan Seidman led to the important discovery of the first mutation in the *MYH7* gene-encoding β-cardiac myosin heavy chain (MHC) [17,20]. Since their seminal work, over 1500 different disease-causing variants have been reported. Mutations in *MYH7* and *MYBPC3*, which encodes myosin binding protein C, account for more than 50% of HCM patients with pathogenic variants [15]. In addition, clinical and genetic studies have supported the causal, albeit relatively uncommon, role of pathogenic variants in other sarcomere protein genes including cardiac troponin T (*TNNT2*), cardiac troponin I (*TNNI3*), α-tropomyosin (*TPM1*), myosin regulatory light chain 2 (*MYL2*), myosin essential light chain (*MYL3*), and actin (*ACTC1*) in HCM [21,22]. Among the known HCM genes, the majority of pathogenic or likely pathogenic variants are predominantly missense, resulting in a nonsynonymous amino acid substitution and suggesting a dominant negative effect [23]. By contrast, most variants in *MYBPC3* are nonsense, caused by frameshifts, splice-site variants, insertions, or deletions that result in a premature stop codon and truncated protein, suggesting a loss of function and haploinsufficiency as an uncommon mechanism in HCM [24]. 

### 3.3. Beyond the Sacomere Genes

**Figure 1:**
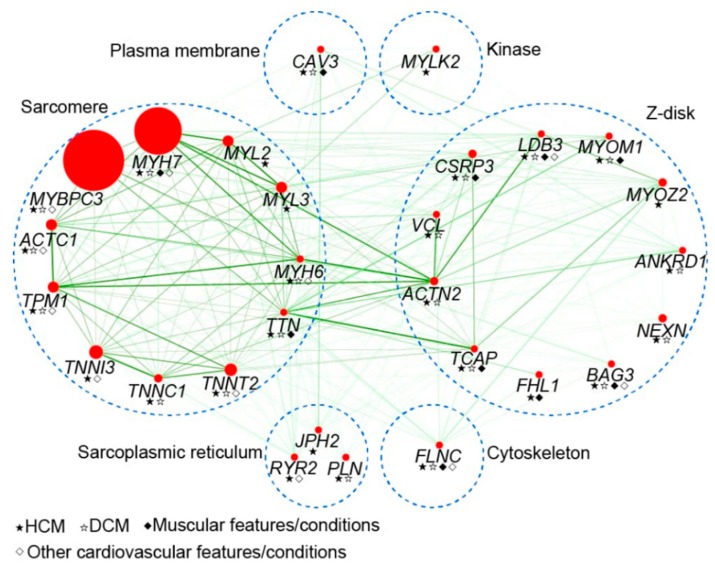
Representation of protein interaction map for genes implicated in hypertrophic cardiomyopathy (HCM). The size of each node (red) is proportional to the relative contribution of each gene to all genotype-positive HCM cases. Predicted interactions are indicated by green color, and the intensity represents correlation score (combined score) between two genes from STRING database (https://string-db.org). The blue dashed circles represent groups of protein classified by the location or role of genes. HCM, hypertrophic cardiomyopathy. DCM, dilated cardiomyopathy.

Due to limited evidence, the clinical significance of other genes has not yet been completely elucidated. These genes include titin (*TTN*), α-actinin (*ACTN1*), α-MHC (*MYH6*), cysteine and glycine rich protein 3 (*CSRP3*), telethonin (*TCAP*), and vinculin (*VCL*) [25,26,27,28,29,30]. Despite occurrences in only isolated cases and small families, variants in myozenin 2 (*MYOZ2*), ubiquitin E3 ligase tripartite motif protein 63 (*TRIM63*), and four-and-a-half LIM domains 1 (*FHL1*), have been reported as causes of HCM (Figure 1) [3,31,32].

Accumulating evidence has supported the role of Ca^2+^ and Na^2+^ homeostasis in the pathogenesis of HCM, that is characterized by the complex interplay between pathogenetic consequences from causal sarcomere mutations and the altered expression and function of ion channels [33]. The abnormal intracellular Ca^2+^ handling has been implicated in downstream cellular arrhythmogenesis in human HCM. Recent studies demonstrated that Ca^2+^ handling is dysregulated via both sarcomere mutation-specific and common pathways [34]. A reduction in the voltage-gated Na^2+^ channel α subunit (SCN5A) full-length mRNA due to abnormal splicing and the subsequent activation of unfolded protein response, a cellular adaptive stress response, has been suggested to contribute to the risk of arrhythmia in HCM patients with sarcomere mutations [35]. In addition, several studies have identified rare genetic variants in genes encoding Ca^2+^ regulatory and handling proteins in HCM, including phospholamban (*PLN*) [36,37], ryanodine receptor 2 (*RYR2*) [38], troponin C (*TNNC1*) [39], and junctophilin 2 (*JPH2*) [40]. 

For most HCM genes, both familial and de novo pathogenic variants have been identified. Recently, the clinical genetic testing of probands with HCM showed that the rate of novel variant detection remained at 35%–40%, with 56% of variants observed in only one family and considered “private” variants [22]. Haplotype analyses of unrelated patients with an identical variant showed that such genetic variants arise independently [41]. In contrast, many unrelated *MYBPC* variant carriers share the same common haplotype architecture in some homogeneous subpopulations including those of Finland, the Netherlands, Japan, India, Iceland and Lebanon, demonstrating clear founder effects in HCM [42,43,44,45,46,47]. Due to benign or late-onset presentations and adverse events, the presence of disease-associated founding mutations or variants in such populations reflects neutral or mild negative selection, but can be associated with an increased risk of HF [43].

Although HCM is considered a predominantly monogenic disease, there is a growing number of roles accounting for phenotypic diversity such as modifying gene variants, epigenetics and other regulatory mechanisms of gene expression, and environmental factors. Despite the recent advances in genetic technologies, causality cannot be unequivocally ascertained, particularly in sporadic cases or small families. Due to extreme genetic diversity, the disease-causing genes remain unknown in ~40% of HCM patients [48]. A subset of small but clinically significant HCM patients (~5%–7%) is found to be attributable to digenic or oligogenic heterozygosity [14]. These findings may not fit with the definition of a classic single gene disease, and raise the possibility that several variants would collectively cause HCM phenotypes, whereas each variant only exerts a mild to moderate effect size. Using NGS technologies, complex genotypes including compound heterozygous or homozygous variants have recently been reported [49]. Besides rare pathogenic gene variants of sarcomere protein, relatively common variants associated with HCM are increasingly recognized [42,43]. For example, other non-sarcomere polymorphisms in genes encoding components of the renin-angiotensin-aldosterone system have been reported to modify the clinical phenotype of HCM [50]. Recently, a pathogenic variant in the mitochondrial genome has been described in families with maternally inherited HCM, attesting to non-sarcomere contributions in HCM pathogenesis [51].

Exercise, diet, cardiac loading conditions, environmental exposures and other illnesses are nongenetic factors that transform the expanding modern landscape of HCM. However, the underlying mechanisms have not yet been precisely characterized. Taken collectively, it underscores the importance of the complex interplay between genetic and nongenetic factors in HCM, and extensive research is ongoing to address the conundrum behind the heterogeneous phenotypes and natural history of HCM.

### 3.4. HCM Phenocopy

Variants in genes associated with LVH can cause a phenotype mimicking HCM (Table 1), accounting for approximately 3% of probands with positive HCM genetic testing [22]. These conditions—that are called HCM phenocopies—include glycogen storage disorders (e.g., Danon disease), protein kinase adenosine monophosphate-activated non-catalytic subunit gamma 2 (PRKAG2) cardiomyopathy, lysosomal storage disorders (e.g., Fabry disease), cardiac amyloidosis, and other inborn errors of metabolism. Despite being relatively less common, it is important to differentiate sarcomeric HCM from these conditions because their management and prognosis greatly differ. Hence, a HCM phenocopy should be considered in patients with unexplained LVH not meeting the HCM diagnostic criteria [5].

## 4. Genetic Testing

Insights into the molecular genetic basis of HCM and advanced sequencing technology have allowed the more feasible, gene-based diagnosis of HCM. Genetic testing of HCM previously relied on polymerase chain reaction (PCR) amplification and Sanger sequencing of amplicons of HCM genes [22]. Some clinical laboratories subsequently developed the gene chip platform using oligo hybridization-based sequencing technology [22,52]. Recently, NGS technologies have been widely adopted, hence NGS-based HCM genetic testing has become more available in both academic and commercial settings. Patients in whom the diagnosis of HCM is established, or suspected, should undergo clinical genetic testing that has been included as a reasonable approach to the diagnosis of HCM [5]. If an index case tests positive for a pathogenic or likely pathogenic variant, cascade screening in family members is recommended. The genetic diagnosis of HCM enables the accurate identification of preclinical variant carriers (genotype-positive/phenotype-negative) which warrant clinical evaluation and surveillance. At-risk individuals who have genotype-negative results no longer require routine electrocardiography and echocardiography. In addition, genetic testing allows for the appropriate reclassification of patient subsets with unrecognized phenocopy conditions such as Fabry disease, where enzyme replacement therapy can halt disease progression and complication [22].

Recently, genotype–phenotype correlations have shown that genotype-positive patients were more severely affected than genotype-negative patients with regard to age at diagnosis, family history of HCM or SCD, and maximum LV wall thickness [53]. Findings from a large multicentre cohort demonstrated that the presence of a sarcomere mutation is associated with earlier disease onset, and serves as a strong predictor of adverse clinical outcomes, including ventricular arrhythmia and HF [54]—underscoring the importance of genotypes in assessing the prognosis of patients and directing clinical management in HCM. Although genetic testing provides a definitive molecular diagnosis and could potentially reduce medical costs from serial clinical evaluations, there are some challenges in current genetic testing for HCM [55]. A number of genes commonly included in HCM gene panels may currently have insufficient evidence of disease association, thus well-validated gene panels are fundamentally critical to avoid genetic misdiagnosis. The failure to identify disease-causing variants in ~50% of patients with HCM (genotype-negative) via clinical genetic testing may be due to undiscovered genetic etiology, potential somatic variants, or acquired cardiomyopathy mimicking HCM. It is important to address the inability to identify a genetic etiology in this clinically important group of HCM, in order to maximize the full potential of genetic testing in HCM. 

## 5. Current and Emerging Therapies

The current clinical management of HCM focuses on two major aspects: (1) symptom management, and (2) risk assessment and the prevention of SCD [5]. Pharmacologic therapy remains the cornerstone of treatment for alleviating HCM symptoms, including using β-adrenergic receptor blocker to blunt the heart rate, increase diastolic filling time, restore cardiac filling pressure and attenuate exercise-induced LVOTO. Disopyramide may be added to reduce symptoms in patients with LVOTO via its negative inotropic effect [56]. L-type Ca^2+^ channel blockers may be used in patients who do not tolerate beta blockers [57]. Diuretics are not normally recommended, though their judicious use may relieve congestive symptoms in select patients.

Despite optimal medical therapy, symptomatic patients with obstructive physiology may benefit from septal reduction therapy, either by septal myectomy or alcohol septal ablation [58]. Rarely, patients with advanced pathology may develop treatment refractory symptoms and decompensation that warrant left ventricular assist device placement or cardiac transplantation. The risk for SCD can range from 0.5%–2% per year in HCM, with ventricular fibrillation being a major cause of arrhythmic death [59]. Placement of an implantable cardioverter-defibrillator (ICD) is recommended in patients who have a high risk for SCD [60]. However, the risk estimation may be imprecise. Recently, extensive myocardial fibrosis detected by contrast-enhanced cardiovascular magnetic resonance imaging with late gadolinium enhancement has been associated with an increased risk for SCD, holding promise for SCD stratification [61]. The novel algorithm which provides estimates of the risk for SCD at 5 years has recently been developed and largely incorporated into clinical practice; however, the definite decision to utilize an ICD in intermediate-risk patients remains challenging [62].

Despite alleviating symptoms, current medical therapies are ineffective in preventing cardiac pathology and complications in HCM. Discoveries in the past decades have elucidated the pathogenetic mechanisms, presenting the opportunity to develop and test several novel treatments to target genetic variants or downstream signals. Preclinical strategies to directly target either damaging missense or loss-of-function variants have been studied in animal models of HCM [63]. These approaches capitalized on viral-mediated gene replacement therapies, or mutation silencing therapies such as short-interfering RNAs [64]. Another emerging target therapy is a small-molecule myosin ATPase inhibitor (MYK-461), that was demonstrated to reduce and prevent the development of HCM pathologies including LVH, myocyte disarray and cardiac fibrosis when orally administered in HCM mice [65]. Remarkably, MYK-461 therapy showed normalization of profibrotic and mitochondrial gene expression. Most recent studies demonstrated that MYK-461 could modulate the myosin interacting-heads motif, a highly conserved sarcomeric structure in which myosin heads bind back onto the thick filament, reducing the numbers of myosins available for cross-bridge formation. As a result, it sequesters myosin heads from their disordered relaxed state, which is a result of HCM variants, to a super relaxed state, reducing hypercontractility and improving relaxation in cardiomyocytes [66]. An open-label trial of MYK-461 (mavacamten) conducted in 11 patients with symptomatic, obstructive HCM for 12 weeks resulted in significant reduction in both resting and post-exercise peak LVOT gradient, and an improvement in NYHA functional class, exercise capacity as assessed by peak oxygen uptake, and levels of NT-proBNP—a biomarker of ventricular wall stress [67]. While the role of genetic or genomic guidance for therapeutic decisions in HCM remains to be precisely defined, the results of multiple ongoing MYK-461 clinical trials are eagerly awaited.

## 6. Conclusions

Over the past several decades, scientific advancements have provided us with unprecedented opportunities to define the genetic landscape of HCM and propelled insights into the key molecular pathways involved in underlying pathogenesis leading to the full range of clinical phenotypes. The emergence of NGS technologies has led to an expansion in the list of variants and genes implicated in HCM. Such knowledge allows the precise identification of at-risk individuals prior to clinical diagnosis, and enables the rapid transformation of the clinical care of individuals with HCM through genetic testing. Moreover, these advances have enabled us to dissect fundamental disease mechanisms involved in sarcomere biology, leading to novel therapeutic approaches for disease modulation and prevention in HCM.

## Figures and Tables

**Table 1:** Summary of genes causing HCM phenocopy conditions.

Gene	Inheritance	Protein Class (HPRD)	Disorders and Associated Phenotypes
*mtDNA*	Mi	Mitochondrial protein	Kearns–Sayre syndrome and multisystem involvement
*BRAF*	AD	Serine/threonine kinase	Noonan syndrome, LEOPARD syndrome, Cardiofaciocutaneous syndrome
*FXN*	AR	Transport/cargo protein	Friedreich ataxia
*GAA*	AR	Enzyme: Glucosidase	Pompe disease
*GLA*	XL	Enzyme: Galactosidase	Fabry disease
*HRAS*	AD	GTPase	Costello syndrome, Congenital myopathy with excess of muscle spindles
*KRAS*	AD	GTPase	Noonan syndrome, Cardiofaciocutaneous syndrome
*LAMP2*	XL	Adhesion molecule	Danon disease
*MAP2K1*	AD	Dual specificity kinase	Cardiofaciocutaneous syndrome
*MAP2K2*	AD	Dual specificity kinase	Cardiofaciocutaneous syndrome
*NRAS*	AD	GTPase	Noonan syndrome
*PRKAG2*	AD	Serine/threonine kinase	Hypertrophic cardiomyopathy (HCM), Wolff–Parkinson–White syndrome, glycogen storage disease of heart
*PTPN11*	AD	Tyrosine phosphatase	Noonan syndrome
*RAF1*	AD	Serine/threonine kinase	LEOPARD syndrome, Noonan syndrome
*RIT1*	AD	GTPase	Noonan syndrome
*SOS1*	AD	Guanine nucleotide exchange factor	Noonan syndrome
*SOS2*	AD	Guanine nucleotide exchange factor	Noonan syndrome 9
*TTR*	AD	Transport/cargo protein	Hereditary transthyretin amyloidosis,

Abbreviations: AD: autosomal dominant inheritance; AR: autosomal recessive inheritance; XL: X-linked inheritance; Mi: mitochondrial inheritance.

## References

[1] Teekakirikul P., Padera R.F., Seidman J.G., Seidman C.E. (2012). Hypertrophic cardiomyopathy: Translating cellular cross talk into therapeutics. J. Cell Biol..

[2] Maron B.J., Gardin J.M., Flack J.M., Gidding S.S., Kurosaki T.T., Bild D.E. (1995). Prevalence of hypertrophic cardiomyopathy in a general population of young adults: Echocardiographic analysis of 4111 subjects in the CARDIA study. Circulation.

[3] Marian A.J., Braunwald E. (2017). Hypertrophic cardiomyopathy: Genetics, Pathogenesis, Clinical Manifestations, Diagnosis, and Therapy. Circ Res..

[4] Maron B.J., Rowin E.J., Udelson J.E., Maron M.S. (2018). Clinical Spectrum and Management of Heart Failure in Hypertrophic Cardiomyopathy. JACC Hear. Fail..

[5] Gersh B.J., Maron B.J., Bonow R.O., Dearani J.A., Fifer M.A., Link M.S., Naidu S.S., Nishimura R.A., Ommen S.R., Rakowski H. (2011). 2011 ACCF/AHA Guideline for the Diagnosis and Treatment of Hypertrophic Cardiomyopathy. J. Am. Coll. Cardiol..

[6] Maron B.J., Doerer J.J., Haas T.S., Tierney D.M., Mueller F.O. (2009). Sudden deaths in young competitive athletes analysis of 1866 deaths in the united states, 1980-2006. Circulation.

[7] Maron B.J., Shirani J., Poliac L.C., Mathenge R., Roberts W.C., Mueller F.O. (1996). Sudden death in young competitive athletes: Clinical, demographic, and pathological profiles. J. Am. Med. Assoc..

[8] Maron B.J., Wolfson J.K., Epstein S.E., Roberts W.C. (1986). Intramural (“small vessel”) coronary artery disease in hypertrophic cardiomyopathy. J. Am. Coll. Cardiol..

[9] Patten M., Pecha S., Aydin A. (2018). Atrial fibrillation in hypertrophic cardiomyopathy: Diagnosis and considerations for management. J. Atr. Fibrillation.

[10] Noubiap J.J., Bigna J.J., Agbor V.N., Mbanga C., Ndoadoumgue A.L., Nkeck J.R., Kamguia A., Nyaga U.F., Ntusi N.A.B. (2019). Meta-analysis of Atrial Fibrillation in Patients With Various Cardiomyopathies. Am. J. Cardiol..

[11] Maisel W.H., Stevenson L.W. (2003). Atrial fibrillation in heart failure: Epidemiology, pathophysiology, and rationale for therapy. Am. J. Cardiol..

[12] Arad M., Penas-Lado M., Monserrat L., Maron B.J., Sherrid M., Ho C.Y., Barr S., Karim A., Olson T.M., Kamisago M. (2005). Gene mutations in apical hypertrophic cardiomyopathy. Circulation.

[13] Maron B.J. (2002). Hypertrophic cardiomyopathy: A systematic review. J. Am. Med. Assoc..

[14] Ho C.Y., Charron P., Richard P., Girolami F., Van Spaendonck-Zwarts K.Y., Pinto Y. (2015). Genetic advances in sarcomeric cardiomyopathies: State of the art. Cardiovasc. Res..

[15] Teekakirikul P., Kelly M.A., Rehm H.L., Lakdawala N.K., Funke B.H. (2013). Inherited cardiomyopathies: Molecular genetics and clinical genetic testing in the postgenomic era. J. Mol. Diagnostics.

[16] Richard P., Charron P., Carrier L., Ledeuil C., Cheav T., Pichereau C., Benaiche A., Isnard R., Dubourg O., Burban M. (2003). Hypertrophic cardiomyopathy: Distribution of disease genes, spectrum of mutations, and implications for a molecular diagnosis strategy. Circulation.

[17] Seidman C.E., Seidman J.G. (2011). Identifying sarcomere gene mutations in hypertrophic cardiomyopathy: A personal history. Circ. Res..

[18] Elliott P.M., Anastasakis A., Borger M.A., Borggrefe M., Cecchi F., Charron P., Hagege A.A., Lafont A., Limongelli G., Mahrholdt H. (2014). European Society of Cardiology Guidelines on diagnosis and management of hypertrophic cardiomyopathy. Eur. Heart J..

[19] Solaro R.J. (2010). Sarcomere control mechanisms and the dynamics of the cardiac cycle. J. Biomed. Biotechnol..

[20] Geisterfer-Lowrance A.A.T., Kass S., Tanigawa G., Vosberg H.P., McKenna W., Seidman C.E., Seidman J.G. (1990). A molecular basis for familial hypertrophic cardiomyopathy: A β cardiac myosin heavy chain gene missense mutation. Cell.

[21] Walsh R., Thomson K.L., Ware J.S., Funke B.H., Woodley J., McGuire K.J., Mazzarotto F., Blair E., Seller A., Taylor J.C. (2017). Reassessment of Mendelian gene pathogenicity using 7,855 cardiomyopathy cases and 60,706 reference samples. Genet. Med..

[22] Alfares A.A., Kelly M.A., McDermott G., Funke B.H., Lebo M.S., Baxter S.B., Shen J., McLaughlin H.M., Clark E.H., Babb L.J. (2015). Results of clinical genetic testing of 2,912 probands with hypertrophic cardiomyopathy: Expanded panels offer limited additional sensitivity. Genet. Med..

[23] Konno T., Chang S., Seidman J.G., Seidman C.E. (2010). Genetics of Hypertrophic cardiomyopathy. Curr. Opin. Cardiol..

[24] Ito K., Patel P.N., Gorham J.M., McDonough B., DePalma S.R., Adler E.E., Lam L., MacRae C.A., Mohiuddin S.M., Fatkin D. (2017). Identification of pathogenic gene mutations in LMNA and MYBPC3 that alter RNA splicing. Proc. Natl. Acad. Sci. U. S. A..

[25] Niimura H., Patton K.K., McKenna W.J., Soults J., Maron B.J., Seidman J.G., Seidman C.E. (2002). Sarcomere protein gene mutations in hypertrophic cardiomyopathy of the elderly. Circulation.

[26] Satoh M., Takahashi M., Sakamoto T., Hiroe M., Marumo F., Kimura A. (1999). Structural analysis of the titin gene in hypertrophic cardiomyopathy: Identification of a novel disease gene. Biochem. Biophys. Res. Commun..

[27] Chiu C., Bagnall R.D., Ingles J., Yeates L., Kennerson M., Donald J.A., Jormakka M., Lind J.M., Semsarian C. (2010). Mutations in Alpha-Actinin-2 Cause Hypertrophic Cardiomyopathy. A Genome-Wide Analysis. J. Am. Coll. Cardiol..

[28] Geier C., Perrot A., Özcelik C., Binner P., Counsell D., Hoffmann K., Pilz B., Martiniak Y., Gehmlich K., Van der Ven P.F.M. (2003). Mutations in the human muscle LIM protein gene in families with hypertrophic cardiomyopathy. Circulation.

[29] Bos J.M., Poley R.N., Ny M., Tester D.J., Xu X., Vatta M., Towbin J.A., Gersh B.J., Ommen S.R., Ackerman M.J. (2006). Genotype-phenotype relationships involving hypertrophic cardiomyopathy-associated mutations in titin, muscle LIM protein, and telethonin. Mol. Genet. Metab..

[30] Vasile V.C., Ommen S.R., Edwards W.D., Ackerman M.J. (2006). A missense mutation in a ubiquitously expressed protein, vinculin, confers susceptibility to hypertrophic cardiomyopathy. Biochem. Biophys. Res. Commun..

[31] Friedrich F.W., Wilding B.R., Reischmann S., Crocini C., Lang P., Charron P., Müller O.J., Mcgrath M.J., Vollert I., Hansen A. (2012). Evidence for FHL1 as a novel disease gene for isolated hypertrophic cardiomyopathy. Hum. Mol. Genet..

[32] Osio A., Tan L., Chen S.N., Lombardi R., Nagueh S.F., Shete S., Roberts R., Willerson J.T., Marian A.J. (2007). Myozenin 2 is a novel gene for human hypertrophic cardiomyopathy. Circ. Res..

[33] Coppini R., Ferrantini C., Mugelli A., Poggesi C., Cerbai E. (2018). Altered Ca2+ and Na+ homeostasis in human hypertrophic cardiomyopathy: Implications for arrhythmogenesis. Front. Physiol..

[34] Helms A.S., Alvarado F.J., Yob J., Tang V.T., Pagani F., Russell M.W., Valdivia H.H., Day S.M. (2016). Genotype-Dependent and -Independent Calcium Signaling Dysregulation in Human Hypertrophic Cardiomyopathy. Circulation.

[35] Noyes A.M., Zhou A., Gao G., Gu L., Day S., Andrew Wasserstrom J., Dudley S.C. (2017). Abnormal sodium channel mRNA splicing in hypertrophic cardiomyopathy. Int. J. Cardiol..

[36] Chiu C., Tebo M., Ingles J., Yeates L., Arthur J.W., Lind J.M., Semsarian C. (2007). Genetic screening of calcium regulation genes in familial hypertrophic cardiomyopathy. J. Mol. Cell. Cardiol..

[37] Landstrom A.P., Adekola B.A., Bos J.M., Ommen S.R., Ackerman M.J. (2011). PLN-encoded phospholamban mutation in a large cohort of hypertrophic cardiomyopathy cases: Summary of the literature and implications for genetic testing. Am. Heart J..

[38] Landstrom A.P., Ackerman M.J. (2012). Beyond the Cardiac Myofilament: Hypertrophic Cardiomyopathy- Associated Mutations in Genes that Encode Calcium-Handling Proteins. Curr. Mol. Med..

[39] Landstrom A.P., Parvatiyar M.S., Pinto J.R., Marquardt M.L., Bos J.M., Tester D.J., Ommen S.R., Potter J.D., Ackerman M.J. (2008). Molecular and functional characterization of novel hypertrophic cardiomyopathy susceptibility mutations in TNNC1-encoded troponin C. J. Mol. Cell. Cardiol..

[40] Landstrom A.P., Weisleder N., Batalden K.B., Martijn Bos J., Tester D.J., Ommen S.R., Wehrens X.H.T., Claycomb W.C., Ko J.K., Hwang M. (2007). Mutations in JPH2-encoded junctophilin-2 associated with hypertrophic cardiomyopathy in humans. J. Mol. Cell. Cardiol..

[41] Saltzman A.J., Mancini-Dinardo D., Li C., Chung W.K., Ho C.Y., Hurst S., Wynn J., Care M., Hamilton R.M., Seidman G.W. (2010). Short communication: The cardiac myosin binding protein C Arg502Trp mutation: A common cause of hypertrophic cardiomyopathy. Circ. Res..

[42] Adalsteinsdottir B., Teekakirikul P., Maron B.J., Burke M.A., Gudbjartsson D.F., Holm H., Stefansson K., DePalma S.R., Mazaika E., McDonough B. (2014). Nationwide study on hypertrophic cardiomyopathy in iceland evidence of a MYBPC3 founder mutation. Circulation.

[43] Dhandapany P.S., Sadayappan S., Xue Y., Powell G.T., Rani D.S., Nallari P., Rai T.S., Khullar M., Soares P., Bahl A. (2009). A common MYBPC3 (cardiac myosin binding protein C) variant associated with cardiomyopathies in South Asia. Nat. Genet..

[44] Jääskeläinen P., Kuusisto J., Miettinen R., Kärkkäinen P., Kärkkäinen S., Heikkinen S., Peltola P., Pihlajamäki J., Vauhkonen I., Laakso M. (2002). Mutations in the cardiac myosin-binding protein C gene are the predominant cause of familial hypertrophic cardiomyopathy in eastern Finland. J. Mol. Med..

[45] Alders M., Jongbloed R., Deelen W., Van den Wijngaard A., Doevendans P., Ten Cate F., Regitz-Zagrosek V., Vosberg H.P., Van Langen I., Wilde A. (2003). The 2373insG mutation in the MYBPC3 gene is a founder mutation, which accounts for nearly one-fourth of the HCM cases in the Netherlands. Eur. Heart J..

[46] Kubo T., Kitaoka H., Okawa M., Matsumura Y., Hitomi N., Yamasaki N., Furuno T., Takata J., Nishinaga M., Kimura A. (2005). Lifelong left ventricular remodeling of hypertrophic cardiomyopathy caused by a founder frameshift deletion mutation in the cardiac myosin-binding protein C gene among Japanese. J. Am. Coll. Cardiol..

[47] Moolman-Smook J.C., De Lange W.J., Bruwer E.C.D., Brink P.A., Corfield V.A. (1999). The origins of hypertrophic cardiomyopathy-causing mutations in two South African subpopulations: A unique profile of both independent and founder events. Am. J. Hum. Genet..

[48] Marian A.J. (2010). Hypertrophic cardiomyopathy: From genetics to treatment. Eur. J. Clin. Invest..

[49] Alpert N.R., Mohiddin S.A., Tripodi D., Jacobson-Hatzell J., Vaughn-Whitley K., Brosseau C., Warshaw D.M., Fananapazir L. (2005). Molecular and phenotypic effects of heterozygous, homozygous, and compound heterozygote myosin heavy-chain mutations. Am. J. Physiol. - Hear. Circ. Physiol..

[50] van der Merwe L., Cloete R., Revera M., Heradien M., Goosen A., Corfield V.A., Brink P.A., Moolman-Smook J.C. (2008). Genetic variation in angiotensin-converting enzyme 2 gene is associated with extent of left ventricular hypertrophy in hypertrophic cardiomyopathy. Hum. Genet..

[51] Govindaraj P., Khan N.A., Rani B., Rani D.S., Selvaraj P., Jyothi V., Bahl A., Narasimhan C., Rakshak D., Premkumar K. (2014). Mitochondrial DNA variations associated with hypertrophic cardiomyopathy. Mitochondrion.

[52] Teekakirikul P., Cox S., Funke B., Rehm H.L. (2011). Targeted sequencing using Affymetrix CustomSeq arrays. Curr. Protoc. Hum. Genet..

[53] Murphy S.L., Anderson J.H., Kapplinger J.D., Kruisselbrink T.M., Gersh B.J., Ommen S.R., Ackerman M.J., Bos J.M. (2016). Evaluation of the Mayo Clinic Phenotype-Based Genotype Predictor Score in Patients with Clinically Diagnosed Hypertrophic Cardiomyopathy. J. Cardiovasc. Transl. Res..

[54] Ho C.Y., Day S.M., Ashley E.A., Michels M., Pereira A.C., Jacoby D., Cirino A.L., Fox J.C., Lakdawala N.K., Ware J.S. (2018). Genotype and Lifetime Burden of Disease in Hypertrophic Cardiomyopathy: Insights from the Sarcomeric Human Cardiomyopathy Registry (SHaRe). Circulation.

[55] Ingles J., Goldstein J., Thaxton C., Caleshu C., Corty E.W., Crowley S.B., Dougherty K., Harrison S.M., McGlaughon J., Milko L.V. (2019). Evaluating the Clinical Validity of Hypertrophic Cardiomyopathy Genes. Circ. Genomic Precis. Med..

[56] Sherrid M.V., Barac I., McKenna W.J., Elliott P.M., Dickie S., Chojnowska L., Casey S., Maron B.J. (2005). Multicenter study of the efficacy and safety of disopyramide in obstructive hypertrophic cardiomyopathy. J. Am. Coll. Cardiol..

[57] Gilligan D.M., Chan W.L., Joshi J., Clarke P., Fletcher A., Krikler S., Oakley C.M. (1993). A double-blind, placebo-controlled crossover trial of nadolol and verapamil in mild and moderately symptomatic hypertrophic cardiomyopathy. J Am Coll Cardiol..

[58] Nishimura R.A., Seggewiss H., Schaff H.V. (2017). Hypertrophic obstructive cardiomyopathy: Surgical myectomy and septal ablation. Circ. Res..

[59] Elliott P.M., Gimeno J.R., Thaman R., Shah J., Ward D., Dickie S., Esteban M.T.T., McKenna W.J. (2006). Historical trends in reported survival rates in patients with hypertrophic cardiomyopathy. Heart.

[60] Maron B.J., Shen W.K., Link M.S., Epstein A.E., Almquist A.K., Daubert J.P., Bardy G.H., Favale S., Rea R.F., Boriani G. (2000). Efficacy of implantable cardioverter-defibrillators for the prevention of sudden death in patients with hypertrophic cardiomyopathy. N. Engl. J. Med..

[61] Chan R.H., Maron B.J., Olivotto I., Pencina M.J., Assenza G.E., Haas T., Lesser J.R., Gruner C., Crean A.M., Rakowski H. (2014). Prognostic value of quantitative contrast-enhanced cardiovascular magnetic resonance for the evaluation of sudden death risk in patients with hypertrophic cardiomyopathy. Circulation.

[62] O’Mahony C., Jichi F., Pavlou M., Monserrat L., Anastasakis A., Rapezzi C., Biagini E., Gimeno J.R., Limongelli G., McKenna W.J. (2014). A novel clinical risk prediction model for sudden cardiac death in hypertrophic cardiomyopathy (HCM Risk-SCD). Eur. Heart J..

[63] Repetti G.G., Toepfer C.N., Seidman J.G., Seidman C.E. (2019). Novel Therapies for Prevention and Early Treatment of Cardiomyopathies. Circ. Res..

[64] Jiang J., Wakimoto H., Seidman J.G., Seidman C.E. (2013). Allele-specific silencing of mutant Myh6 transcripts in mice suppresses hypertrophic cardiomyopathy. Science.

[65] Green E.M., Wakimoto H., Anderson R.L. (2016). A small-molecule inhibitor of sarcomere contractility suppresses hyperthrophic cardiomyopathy in mice. Science.

[66] Anderson R.L., Trivedi D.V., Sarkar S.S., Henze M., Ma W., Gong H., Rogers C.S., Gorham J.M., Wong F.L., Morck M.M. (2018). Deciphering the super relaxed state of human β-cardiac myosin and the mode of action of mavacamten from myosin molecules to muscle fibers. Proc. Natl. Acad. Sci. U. S. A..

[67] Jacoby D., Lester S., Owens A., Wang A., Young D., Tripuraneni R., Semigran M., Heitner S. (2018). Reduction in left ventricular outflow tract gradient with mavacamten (myk-461) in symptomatic obstructive hypertrophic cardiomyopathy patients (PIONEER-HCM). J. Am. Coll. Cardiol..

